# Codon Distribution in Error-Detecting Circular Codes

**DOI:** 10.3390/life6010014

**Published:** 2016-03-15

**Authors:** Elena Fimmel, Lutz Strüngmann

**Affiliations:** Institute for Mathematical Biology, Faculty of Computer Science, Mannheim University of Applied Sciences, Paul-Wittsack Str. 10, 68163 Mannheim, Germany; l.struengmann@hs-mannheim.de

**Keywords:** genetic code, comma-free codes, circular codes, codon usage, evolution of the genetic code

## Abstract

In 1957, Francis Crick *et al.* suggested an ingenious explanation for the process of frame maintenance. The idea was based on the notion of comma-free codes. Although Crick’s hypothesis proved to be wrong, in 1996, Arquès and Michel discovered the existence of a weaker version of such codes in eukaryote and prokaryote genomes, namely the so-called circular codes. Since then, circular code theory has invariably evoked great interest and made significant progress. In this article, the codon distributions in maximal comma-free, maximal self-complementary $C^{3}$ and maximal self-complementary circular codes are discussed, *i.e.*, we investigate in how many of such codes a given codon participates. As the main (and surprising) result, it is shown that the codons can be separated into very few classes (three, or five, or six) with respect to their frequency. Moreover, the distribution classes can be hierarchically ordered as refinements from maximal comma-free codes via maximal self-complementary $C^{3}$ codes to maximal self-complementary circular codes.

## 1. Introduction

The genetic code as it is today is a product of a long evolutionary process. It can be seen as a kind of dictionary that translates information from the world of nucleic acids into the world of proteins. As such, it is involved in the transmission of information, the translation process, and thus, plays an essential role in the process that defines the central dogma of molecular biology. During this process, degeneracy is one of the most conserved features of the genetic code. It can be postulated that this is for a good reason, since degeneracy is the fundamental ingredient in any error-detecting and error-correcting system [1,2]. Therefore, for example, the self-referential model for the formation of the code is based on an original regionalization of characters through the concerted superposition of the two components of the encodings. With this approach, the degeneracy of the genetic code and clusters of similar amino acids corresponding to similar triplets should be explained [3].

The preservation of the genetic information is impossible without an error-correcting system (this can even be proven by the methods of information theory) and cannot be guaranteed just by DNA replication. There are different hypotheses for how such error-correction may happen. In [4], the so-called ambush hypothesis is examined. According to this hypothesis, off-frame stops terminate frameshifted translation, potentially decreasing energy and resource waste on nonfunctional proteins. Moreover, codons with more potential to form hidden stops (off-frame stops) have greater usage frequency and bias in their favor among synonymous codons. In [5], a model of an amino acid composed of a constant part and of a variable part is considered, and it was concluded that the kinetic energetic disturbance caused by the substitution of the variable part of an amino acid is minimized. In [6], error prevention and mitigation as forces in the evolution of genes and genomes are postulated.

Errors in the translation process can occur in several ways, e.g., mutation of the genetic information or insertion/deletion of nucleotides. In the late 1950s, biologists tripped over another essential source of errors, the so-called *frameshift problem*: a sequence of nucleotides can be translated correctly into a chain of amino acids only when it is read in the *correct* frame. The first reaction to this problem was the new concept of “codes without commas”, nowadays called *comma-free codes*, suggested by Crick, Griffith and Orgel [7]. They hypothesized that only a subset of codons is actually used for the translation. The strong property of such codes is the immediate detection of the wrong reading frame. Each out-of-frame codon in a sequence of codons from a comma-free code is outside the code and has therefore no *meaning*. In particular, the use of a comma-free code would re-establish the correct reading frame within a *window* of three nucleotide bases. Unfortunately, after the discovery of the standard genetic code lexicon by Nirenberg and Matthaei [8] (see also Khorana [9]), it became clear that the elegant theory of Crick *et al.* is not valid in the form proposed. For instance, the trinucleotide TTT, an excluded trinucleotide in a comma-free code, codes phenylalanine [8].

The motivation to study comma-free codes again came after the discovery of so-called circular codes, which are a weaker version of comma-free codes. In Arquès and Michel [10], a set $X_{0}$ of 20 codons was identified by statistical analysis of genes of prokaryotes and eukaryotes. These 20 codons appear preferentially in the correct reading frame and have the property of detecting frame shifts not immediately, but eventually. In fact, at most 13 consecutive nucleotides in a sequence of codons from the code $X_{0}$ are enough to detect the correct reading frame. In 2015, by quantifying the approach used in 1996 and by applying massive statistical analysis of gene taxonomic groups, the circular code detected in 1996 was rediscovered extensively in genes of prokaryotes and eukaryotes and now also identified in the genes of plasmids and viruses [11]. The codes discovered by Arquès and Michel in nature have even more interesting properties. With each codon, its anticodon is also in the code (*self-complementarity*), and they also have the error detection property in frame 1 and 2 (*$C^{3}$-property*). Such codes are called *self-complementary $C^{3}$ codes* and have been completely classified in [10,12] and [13]. A weaker version of such codes are *self-complementary circular codes*, which are self-complementary, but cannot recognize if the reading-frame is shifted by one or two bases. Any comma-free code, self-complementary $C^{3}$-code or self-complementary circular code can contain at most 20 codons (see, e.g., [14]), and all such maximal codes have been completely classified by computer calculations: there are exactly 216 maximal self-complementary $C^{3}$ codes, exactly 408 maximal comma-free codes and exactly 528 maximal self-complementary circular codes (see [10,14,15,16,17,18]). None of the maximal comma-free codes, however, can be self-complementary.

In the present work, we first discuss some *ancient genetic codes*, e.g., the *primeval code*, the RNY code, SNS code and the NNS code, that have been postulated in several theories about the evolution of the genetic code as a predecessor of the current standard genetic code (see [19,20,21,22,23,24]). This is by far not a complete list of such hypothesized codes, but serves as a motivating list of examples based on biological concepts, the RNY code being the most important one, since it has been statistically observed in genes on the two-letter alphabet {R,Y}. We show that all of these ancient genetic codes that used only some of the 64 codons always contain a large comma-free code that codes for almost all of the amino acids involved. This shows that in predecessors of the current genetic code, Crick’s hypothesis on the usage of a comma-free code was much more likely and could have been true. However, nowadays, the genetic code has become too *complex* to use such strong codes (in the sense of having strong error-detecting properties, *i.e.*, recognizing a frameshift immediately), and therefore, nature moved on to the weaker circular codes. Thus, it is very likely that the circular codes have evolved from the comma-free codes in some way. In this article, we give some hints for this hypothesis, which would shed more light on the evolutionary development of the genetic code and why it is as it is.

We consider the three classes of codes: maximal comma-free, maximal self-complementary circular and maximal self-complementary $C^{3}$ codes. For each codon, except for the excluded AAA,CCC,GGG,TTT, we calculate how many codes of the three classes considered it can appear in; this is called the *frequency class number* of the codon with respect to a class of codes. After preparing definitions (see Section 2), the main results of the article are presented in Section 3. Surprisingly, it turns out that for each of the above classes of codes, there are very few frequency class numbers of codons with respect to it (see also [16,17] for the data). It is even more surprising that the frequency classes of codons for self-complementary $C^{3}$ codes are *refinements* of the classes for comma-free codes, and those for self-complementary circular codes are refinements for the corresponding classes of self-complementary $C^{3}$ codes. Thus, the number of different frequency classes of codons increases parallel to the decrease of error-detecting properties of the codes. The fact that the frequency classes of the codons for maximal self-complementary $C^{3}$ codes are refinements of the classes for maximal comma-free codes is a strong hint that the maximal self-complementary $C^{3}$ codes used in the current genetic code could have evolved from the maximal comma-free codes, which were very likely used in earlier times, since these two classes of codes are disjoint. This means that there is no obvious mathematical reason for this refinement property.

Our results strengthen the supposition that the modern codes originated from ancient (self-complementary) comma-free codes (see [23,24]) and, as a consequence, a weaker version of the Crick *et al.* theory.

## 2. Definitions and Notations

The genetic code is written with words of three letters, called *codons*, built over an alphabet:

B:={U(T),C,A,G}

of four letters, nucleotide bases *uracil (thymine), cytosine, adenine* and *guanine*, in short U(T),C,A and G. In recent studies, e.g., [11,12,14,18,25], the structure of certain sub-codes of the genetic code that are assumed to play a role in nature were investigated. The first class of codes was suggested by Crick *et al.* in [7].

**Definition 1.**  A trinucleotide code $X\subseteq B^{3}$ is called comma-free if any given two codons $x_{1},x_{2}\in X$, any sub-codon of the concatenation $x_{1}x_{2}$, except $x_{1},x_{2}$ themselves, does not belong to X. We will call a trinucleotide comma-free code X maximal if it contains exactly 20 codons.

**Figure 1:**
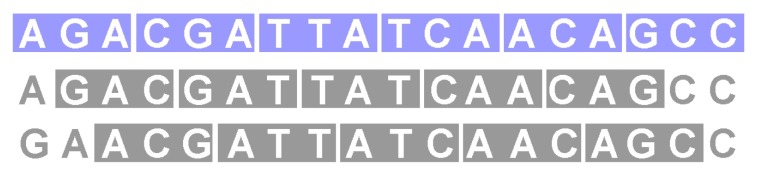
For comma-free codes, a frameshift is detected immediately. All codons highlighted in gray in the second and the third row are not in the code.

Being comma-free means that a frameshift of one or two bases is detected after reading of three nucleotide bases (see Figure 1). We would like to mention at this point that our point of view of the frameshift problem is an information theoretical point of view. In living cells, a frameshift is of course also “detected” because of the mistranslated protein product that is produced and its potential phenotypic consequences.

Clearly, a comma-free code cannot contain the *periodical* codons AAA, CCC, GGG or TTT since, for example, a frame shift in a sequence of adenines could not be detected. Moreover, for any codon $B_{1}B_{2}B_{3}$ from a comma-free code, the *shifted* codons $B_{2}B_{3}B_{1}$ and $B_{3}B_{1}B_{2}$ cannot be in the same code. For instance, if ACG is in the code, then CGA and GAC must not be in the same code, because they appear in frameshift 1 and 2 of the:

ACGACGACGACG...

ACGACGACGACG...

ACGACGACGACG...

The two *shift operations* are commonly denoted by $\alpha_{1}$ and $\alpha_{2}$, *i.e.*, $\alpha_{1}\left(B_{1}B_{2}B_{3}\right)=B_{2}B_{3}B_{1}$ and $\alpha_{2}\left(B_{1}B_{2}B_{3}\right)=B_{3}B_{1}B_{2}$ for any codon $B_{1}B_{2}B_{3}\in B^{3}$ (see, for instance, [12]). Thus, any comma-free code can at most contain one codon out of the three ACG,CGA and GAC and similarly for any other codon. Thus, the maximal number of codons in a comma-free code is $20=\frac{64-4}{3}$, as required in the above Definition 1.

Maximal comma-free codes have been completely classified by computer calculations, and it turned out that there are exactly 408 such codes (see [15]).

At this point, we would like to discuss some examples of *ancient codes*, *i.e.*, genetic code tables that were suggested as a predecessor of the current standard genetic code in some theory about the evolution of the genetic code. These codes coded only for a few amino acids and very often also used only some of the codons and not all. Surprisingly, most of these codes contained a *large* comma-free sub-code that codes for almost all of the amino acids or were even themselves comma-free. Note that any comma-free code can code for at most 13 amino acids ([16]; see also Table 4 in [26]), while a circular code can code for at most 18 amino acids [27].

**Example** **1.**

- *In the generalized co-evolution theory by Di Giulio [20,21], the SNS code (the letter S stands here for the strong nucleotide bases C or G, in contrast to weak nucleotide bases A or T) was suggested and consists of the following codons coding for the seven amino acids valine, glutamine, alanine, asparagine, glycine and serine:*
  $$X_{SNS}=\left\{\begin{array}{cccccccc} \mathrm{CTC}, & \mathrm{CCC}, & \mathrm{CAC}, & \mathrm{CGC}, & \mathrm{CTG}, & \mathrm{CCG}, & \mathrm{CAG}, & \mathrm{CGG},\\ \mathrm{GTC}, & \mathrm{GCC}, & \mathrm{GAC}, & \mathrm{GGC}, & \mathrm{GTG}, & \mathrm{GCG}, & \mathrm{GAG}, & \mathrm{GGG} \end{array}\right\}$$
  *It contains the comma-free sub-code:*
  $$Y_{SNS}=\left\{\mathrm{CTC},\mathrm{GGC},\mathrm{GTC},\mathrm{CTG},\mathrm{CAC},\mathrm{GTG},\mathrm{GAC},\mathrm{CAG},\mathrm{GAG},\mathrm{CGC}\right\}$$
  *which is as large as possible (of size 10), because there are two codons of the form NNN in $X_{SNS}$, and four codons have a cyclically-equivalent codon in the code. It codes for all but one amino acid, namely alanine.*
- *In the generalized co-evolution theory by Di Giulio [20,21], the NNS code was suggested and consists of the following codons coding for the seven amino acids valine, glutamine, alanine, asparagine, glycine and serine plus the stop signal:*
  $$X_{NNS}=\left\{\begin{array}{cccccccc} \mathrm{TTC}, & \mathrm{TCT}, & \mathrm{TAC}, & \mathrm{TGC}, & \mathrm{TCG}, & \mathrm{TAG}, & \mathrm{AGG}, & \mathrm{CTC},\\ \mathrm{CCC}, & \mathrm{CAC}, & \mathrm{CGC}, & \mathrm{CTG}, & \mathrm{CCG}, & \mathrm{CAG}, & \mathrm{CGG}, & \mathrm{ATC},\\ \mathrm{ACC}, & \mathrm{ACA}, & \mathrm{ACG}, & \mathrm{ATG}, & \mathrm{ACG}, & \mathrm{AAG}, & \mathrm{GTC}, & \mathrm{GCC},\\ \mathrm{GAC}, & \mathrm{GGC}, & \mathrm{GTG}, & \mathrm{GCG}, & \mathrm{GAG}, & \mathrm{GGG} \end{array}\right\}$$
  *It contains the comma-free sub-code:*
  $$Y_{NNS}=\left\{\mathrm{CTC},\mathrm{GGC},\mathrm{TTC},\mathrm{GTC},\mathrm{CTG},\mathrm{TAC},\mathrm{GAA},\mathrm{GAC},\mathrm{CAG},\mathrm{GAG},\mathrm{ATC}\right\}$$
  *which is as large as possible (of size 11) and codes for all except one amino acid, namely alanine, and the stop signal.*
- *The RNY code [19] consists of the eight amino acids glycine, threonine, asparagine, serine, valine, arginine, isoleucine and alanine and the 16 codons:*
  $$X_{RNYl}=\left\{\begin{array}{cccccccc} GGT, & GGC, & ACT, & ACC, & AGC, & AGT, & GAC, & GAT,\\ GTC, & GTT, & AAT, & ATT, & AAC, & ATC, & GCT, & GCC \end{array}\right\}$$
  *This code is comma-free.*
- *The theory of Jolivet and Rothen [22] yields another primeval code, which codes for the amino acids tyrosine, alanine, phenylalanine, arginine, valine, asparagine, aspartate, leucine, glutamate, glycine, glutamine and isoleucine:*
  $$X_{primeval}=\left\{\begin{array}{ccccccc} \mathrm{TTC}, & \mathrm{TAC}, & \mathrm{CTC}, & \mathrm{CTG}, & \mathrm{CAG}, & \mathrm{ATC}, & \mathrm{AAC},\\ \mathrm{GTT}, & \mathrm{GTC}, & \mathrm{GTA}, & \mathrm{GCC}, & \mathrm{GAT}, & \mathrm{GAC}, & \mathrm{GAG},\\ \mathrm{GAA}, & \mathrm{GGC} \end{array}\right\}$$
  *The code is even a $C^{3}$ code (see below). Moreover, it contains a comma-free sub-code of size 11 (as large as possible) that codes for all amino acids, except for two, namely alanine and arginine:*
  $$Y_{primeval}=\left\{\mathrm{CTC},\mathrm{GGC},\mathrm{TTC},\mathrm{AAC},\mathrm{GTC},\mathrm{CTG},\mathrm{TAC},\mathrm{GAC},\mathrm{CAG},\mathrm{GAG},\mathrm{ATC}\right\}$$

As mentioned in the Introduction, this list of examples of predecessor codes is by far not complete, and most of these codes are based on biological concepts. The most important code is the RNY code [19], which has also been statistically observed in genes on the two-letter alphabet {R,Y}. It is comma-free, and it was already shown in [10] that the RNY code can be also constructed by looking at the preferential frame of the RNY codons in genes. In fact, in [10], the authors assigned to each codon a preferential frame (which then led to the discovery of the first maximal self-complementary $C^{3}$-code), and taking the average frame of the RNY codons, it was pointed out by the authors that this is in fact Frame 0 (see [10], Table 3a–c and the corresponding discussion).

As we can see, the ancient codes discussed in the above Example 1 always contain a large sub-code that is *comma-free* and encodes *almost all of the amino acids* used in the code. This is impossible in the current standard genetic code, as we have seen above.

Since Crick’s hypothesis on the usage of comma-free codes consequently had to fail in nature, the next class of codes that was investigated is the class of maximal self-complementary $C^{3}$ codes.

**Definition 2.**  We will call a set of codons $X\subseteq B^{3}$ a trinucleotide circular code if any word over the alphabet B written on a circle has at most one decomposition into words from X. By word written on a circle, it is intended that after the last letter, the word starts again (from its first letter). We will call a trinucleotide circular code X maximal if it contains exactly 20 codons.

**Figure 2:**
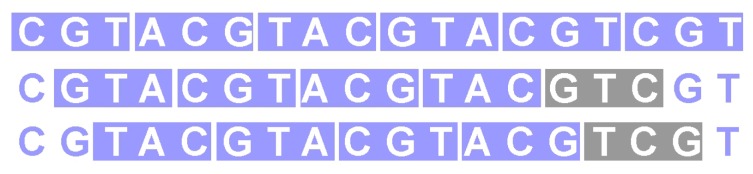
For circular codes, a frameshift is detected after a few codons. The codons highlighted in gray in the second and the third row are not in the code.

Circular codes do not allow the detection a frameshift immediately as comma-free codes do, but eventually, after a few codons (see Figure 2). Thus, it is obvious that any comma-free code is also circular.

The first maximal circular code that was found in nature by Arquès and Michel [10] is:

$$X_{0}=\left\{\begin{array}{cccccccc} \mathrm{AAC}, & \mathrm{AAT}, & \mathrm{ACC}, & \mathrm{ATC}, & \mathrm{ATT}, & \mathrm{CAG}, & \mathrm{CTC}, & \mathrm{CTG},\\ \mathrm{GAA}, & \mathrm{GAC}, & \mathrm{GAG}, & \mathrm{GAT}, & \mathrm{GCC}, & \mathrm{GGC}, & \mathrm{GGT}, & \mathrm{GTA},\\ \mathrm{GTC}, & \mathrm{GTT}, & \mathrm{TAC}, & \mathrm{TTC}\; \end{array}\right\}$$

The code $X_{0}$ had even stronger properties. The first one says that the code is not just error-detecting in the normal reading frame, but also in shifts 1 and 2.

**Definition 3.**  *Let $X\subseteq B^{3}$. We will say that X is a $C^{3}$* code *if X, as well as $X_{1}$ and $X_{2}$ are circular, where $X_{1}:=\alpha_{1}\left(X\right)$ and $X_{2}:=\alpha_{2}\left(X\right)$.*

Moreover, the code found by Arquès and Michel was also *invariant* under forming the anticodons of its members.

**Definition 4.**  *Let $X\subseteq B^{3}$. We will call X self-complementary if with each codon x∈X, its anticodon is also in X.* 

Again, computer calculations showed that there are exactly 528 maximal self-complementary circular codes containing exactly 216 maximal self-complementary $C^{3}$ codes (see [10,13,28]).

In the recent investigations of comma-free and circular codes, the group of permutations (bijective transformations) of bases played a significant role (see, for example, [12,29]). Recall that a *permutation* of the bases from B is just a *bijective shuffling* of the bases. Formally, the symmetric group on the set B is defined as:

$$S_{B}=\{\pi :B\to B|\;\pi \;is\;bijective\}$$

with the group operation of function composition. Bijective transformations *π*: *ℬ* → ℬ can be applied componentwise to *x* ∈ ℬ^3^ and, thus, induce a bijective map ℬ^3^ → ℬ^3^ which we will denote also by *π*. Hence, *π* systematically exchanges bases in a codon or sequence of codons, and there are exactly 24 such transformations. The *complementing map* plays a very essential role:

c:B→B

with

c(A)=T,c(T)=A,c(C)=G,c(G)=C

which assigns to each basis its complementary basis. An important property of permutations is the following (see [12,29]):

(1) Any permutation preserves comma-freeness and circularity, hence the C3 property.

However, self-complementarity is not always preserved, but is by eight of the 24 permutations. These permutations, among which we find the complementing map *c*, were characterized in [12] and form a subgroup of the symmetric group $S_{B}$. Finally, the so-called *reversing permutation*, which reverses a codon, *i.e.*, $\overset{\leftarrow }{\left(B_{1},B_{2},B_{3}\right)}:=\left(B_{3},B_{2},B_{1}\right),B_{i}\in B$, also preserves self-complementarity (see [12]). Note that the anticodon of a codon *x* can be expressed as $\overset{\leftarrow }{c(x)}$. Thus, we have:

(2) If X is a comma-free code, then its reversed code X← and its code of anticodons c(X)← are both comma-free, as well;

(3) If X is a circular self-complementary code, then its reversed code X← and its code of anticodons c(X)← are both circular self-complementary, as well.

We would like to draw the reader to very interesting works by Seligmann [30,31,32,33] that are related to the bijective transformations $S_{B}$. In fact, it was shown by Seligmann that parts of actual DNA and RNA sequences are replicated by systematic exchanges of nucleotides, *i.e.*, by applying one of the 24 bijective transformations to it. These sequences are called *swinger sequences*, and convergence between swinger sequences detected are based on classical PCR sequencing methods.

## 3. Distribution of Codons in Maximal Error-Detecting Codes

In this section, we will mainly consider three classes of error-detecting and error-correcting codes that have appeared in the development and theory of the genetic code: the class COM of all maximal comma-free codes, the class CIRC of all maximal circular self-complementary codes and the class C3 of all maximal self-complementary $C^{3}$ codes. We are interested in the *frequency* of codons appearing in such codes, *i.e.*, we will determine for each codon the number of codes from the above three classes in which it appears. We start with the following:

**Definition 5.**  *Let $x\in B^{3}$ be a codon, and let K be either the class COM of all maximal comma-free codes, or the class CIRC of all maximal circular self-complementary codes, or the class C3 of all maximal self-complementary $C^{3}$ codes. Then:*

$$u_{K}\left(x\right)=|\left\{K\in K:x\in K\right\}|$$

*denotes the number of codes K from the class K, such that x belongs to K. The number $u_{K}\left(x\right)$ is called the frequency class number of x with respect to K.*

A first easy observation is that for any codon *x* and any of the above classes K, the frequency number of *x* with respect to K is the same as the frequency number of the anticodon $\overset{\leftarrow }{c(x)}$ with respect to K, as well as that of the reversed codon $\overset{\leftarrow }{x}$ of *x* with respect to K (for comma-free codes this follows from Equations (2) and (3) above):

(4) $$u_{K}\left(x\right)=u_{K}\left(\overset{\leftarrow }{x}\right)=u_{K}\left(\overset{\leftarrow }{c(x)}\right)$$

Moreover, by the maximality of the codes in all of the above classes, we also have:

(5) $$u_{K}\left(x\right)+u_{K}\left(\alpha_{1}\left(x\right)\right)+u_{K}\left(\alpha_{2}\left(x\right)\right)=|K|$$

for all codons *x* and classes K=COM, K=C3, K=CIRC. Recall that $\alpha_{1}\left(x\right)$ and $\alpha_{2}\left(x\right)$ are the circular permutations of *x*.

We now show all equivalence numbers of codons with respect to the three classes of codes CIRC, C3 and COM. Recall first that there are exactly 408 maximal comma-free codes and that clearly any codon contained in a comma-free code either consists of three different bases or has exactly two identical bases. Thus, the cases in the following theorem cover all possible codons.

**Theorem 6.**  *Let $x\in B^{3}$ be a codon and K=COM the class of all maximal comma-free codes. Then, the following statements are true:*

1. *If $x=B_{1}B_{2}B_{1}$ with $B_{1},B_{2}\in B$ and $B_{1}\neq B_{2}$, then:*
  $$u_{K}\left(x\right)=184$$
2. *If $x=B_{2}B_{1}B_{1}$ or $x=B_{1}B_{1}B_{2}$ with $B_{1},B_{2}\in B$ and $B_{1}\neq B_{2}$, then:*
  $$u_{K}\left(x\right)=112$$
3. *If $x=B_{1}B_{2}B_{3}$ with $B_{1},B_{2},B_{3}\in B$ and $B_{1}\neq B_{2},B_{1}\neq B_{3},B_{2}\neq B_{3}$, then:*
  $$u_{K}\left(x\right)=136$$

*In particular, there are only three different frequency class numbers 112, 136 and 184 for codons with respect to the class COM of all maximal comma-free codes.*

The following Table 1 illustrates the result from the above Theorem 6 showing the frequency class numbers of codons with respect to COM. Recall that the *trivial* codons AAA,CCC,GGG,TTT can never be part of any error-detecting system; hence, there are only 60 codons shown in the next table. Codons colored in the same color have the same frequency class number.

Theorem 6 was obtained by computer calculations, but proofs of some parts of Theorem 6 can be found in Appendix A. However, it is easy to see why there are only three different frequency class numbers. The reason for this is of a group theoretic nature. Since any permutation $\pi \in S_{B}$ carries a maximal comma-free code into a maximal comma-free code, it follows that for any codon $x\in B^{3}$, we have $u_{\mathrm{COM}}\left(x\right)=u_{\mathrm{COM}}\left(\pi \left(x\right)\right)$. Thus, all codons consisting of three different bases must have the same frequency class number with respect to COM. Moreover, those with two identical bases in positions 1 and 3 must have the same frequency class number, and finally, the codons with two identical bases in positions 1 and 2, as well as in positions 2 and 3 must have the same frequency class numbers. The latter follows from Equation (4).

The following Table 2 illustrates the result from Theorem 6 above showing the distribution of codons with respect to COM in the standard genetic code table.

The next theorem gives the same characterization of frequency numbers with respect to the class CIRC of maximal circular self-complementary codes. Note that the number of such codes is exactly 528. Moreover, note that any codon has either two identical bases, and then, the third basis is the complementary one (Cases (1) and (2) in the next theorem), or the third basis is not the complementary one (Cases (3) and (4) below), or the codon has three different bases and in these cases, two of them must be complementary to each other (Cases (5) and (6)). Thus, the case distinction in the following theorem covers all possible codons.

**Theorem 7.**  *Let $x\in B^{3}$ be a codon and K=CIRC the class of all maximal circular self-complementary codes. Then, the following holds:*

1. *If $x=B_{1}c\left(B_{1}\right)B_{1}$ with $B_{1}\in B$, then:*
  $$u_{K}\left(x\right)=0$$
2. *If $x=B_{1}B_{1}c\left(B_{1}\right)$ or $x=c\left(B_{1}\right)B_{1}B_{1}$ with $B_{1}\in B$, then:*
  $$u_{K}\left(x\right)=264$$
3. *If $x=B_{1}B_{1}B_{2}$ or $x=B_{2}B_{1}B_{1}$ with $B_{1},B_{2}\in B$, $B_{1}\neq B_{2},B_{2}\neq c\left(B_{1}\right)$, then:*
  $$u_{K}\left(x\right)=187$$
4. *If $x=B_{1}B_{2}B_{1}$ with $B_{1},B_{2}\in B$, $B_{1}\neq B_{2}$, $B_{2}\neq c\left(B_{1}\right)$, then:*
  $$u_{K}\left(x\right)=154$$
5. *If x=$B_{1}B_{2}c\left(B_{1}\right)$ with $B_{1},B_{2}\in B,B_{1}\neq B_{2},B_{2}\neq c\left(B_{1}\right)$., then:*
  $$u_{K}\left(x\right)=234$$
6. *If $x=B_{1}c\left(B_{1}\right)B_{2}$ or $x=B_{1}c\left(B_{2}\right)B_{2}$ with $B_{1},B_{2}\in B$, $B_{1}\neq B_{2}$, $B_{2}\neq c\left(B_{1}\right)$, then:*
  $$u_{K}\left(x\right)=147$$

*In particular, there are only the six different frequency class numbers,* 0, 147, 154, 187, 234 *and* 264, *for codons with respect to the class CIRC of all maximal circular self-complementary codes.*

The following Table 3 illustrates the result from Theorem 7 above showing the frequency class numbers of codons with respect to CIRC. Again, recall that the *trivial* codons AAA,CCC,GGG,TTT can never be part of any error-detecting system; hence, there are only 60 codons shown in the next table.

As for Theorem 6, the results of Theorem 7 were found by computer calculations, but a mathematical proof of some parts of the theorem can be found in Appendix B. Again, group theory shows why there are only a few different frequency class numbers. However, this time, there are only eight transformations π∈B that carry self-complementary circular codes into self-complementary circular codes. These eight transformations were classified as the dihedral group *L* in [12]. Thus, for these eight transformations and any codon $x\in B^{3}$, we have $u_{\mathrm{CIRC}}\left(x\right)=u_{\mathrm{CIRC}}\left(\pi \left(x\right)\right)$. Thus, all codons consisting of three different bases must have the same frequency class number with respect to CIRC if they can be mapped onto each other by a permutation from *L*. The same holds for those codons with two identical bases in two out of the three positions.

The following Table 4 illustrates the result from Theorem 7 above showing the distribution of codons with respect to CIRC in the standard genetic code table.

A surprising fact is that the frequency class numbers of codons with respect to the class CIRC of all maximal circular self-complementary codes is a refinement of the frequency class numbers of codons with respect to the class COM of all maximal comma-free codes. This is not at all clear, since the two classes COM and CIRC are disjoint. No maximal comma-free code is self-complementary. We will come back to this point after the next theorem and its illustration.

We finally show the frequency class numbers of codons with respect to the class C3 of all maximal self-complementary $C^{3}$ codes. Note that the number of such codes is exactly 216. Moreover, note that as above, the case distinction in the following theorem covers all possible codons.

**Theorem 8.**  *Let $x\in B^{3}$ be a codon and K=C3 the class of all maximal self-complementary $C^{3}$ codes. Then, the following holds:*

1. *If $x=B_{1}B_{1}c\left(B_{1}\right)$ or $x=c\left(B_{1}\right)B_{1}B_{1}$ with $B_{1}\in B$, then:*
  $$u_{K}\left(x\right)=108$$
2. *If $x=B_{1}c\left(B_{1}\right)B_{1}$ with $B_{1}\in B$, then:*
  $$u_{K}\left(x\right)=0$$
3. *If $x=B_{1}B_{1}B_{2}$ or $x=B_{2}B_{1}B_{1}$ or $x=B_{1}B_{2}B_{1}$ with $B_{1},B_{2}\in B$, $B_{1}\neq B_{2}$, $B_{2}\neq c\left(B_{1}\right)$, then:*
  $$u_{K}\left(x\right)=72$$
4. *If $x=B_{1}B_{2}c\left(B_{1}\right)$ with $B_{1},B_{2}\in B$, $B_{1}\neq B_{2}$, $B_{2}\neq c\left(B_{1}\right)$, then:*
  $$u_{K}\left(x\right)=98$$
5. *If $x=B_{1}c\left(B_{1}\right)B_{2}$ or $x=B_{1}c\left(B_{2}\right)B_{2}$ with $B_{1},B_{2},B_{3}\in B$, $B_{1}\neq B_{2}$, $B_{2}\neq c\left(B_{1}\right)$, then:*
  $$u_{K}\left(x\right)=59$$

*In particular, there are only the five different frequency class numbers,* 0, 59, 72, 98 *and* 108, *for codons with respect to the class C3 of all maximal self-complementary $C^{3}$ codes.*

As for Theorems 6 and 7, the results in Theorem 8 were discovered by computer calculation; however, some parts of the above theorem are proven in Appendix C. As for Theorem 7, the action of the dihedral group *L* on the set C3 explains why there are only a few different frequency classes.

The following Table 5 and Table 6 illustrate the result from the above Theorem 8 showing the frequency class numbers of codons with respect to C3 and their distribution in the standard genetic code table. Recall once more that the *trivial* codons AAA,CCC,GGG,TTT can never be part of any error-detecting system; hence, there are only 60 codons shown in the next table.

Since the maximal self-complementary $C^{3}$ codes are a subset of the set of maximal circular self-complementary codes, it is clear that the splitting of codons with respect to their frequency class numbers cannot be significantly different. Nevertheless, as we can see, it is not completely the same. The Classes (3) and (4) from Theorem 7 merge to one class in Theorem 8 (Class (3)), due to the additional $C^{3}$-property. Thus, the frequency class numbers of codons with respect to the class CIRC is a refinement of the ones with respect to the class C3.

## 4. Results, Discussion and Conclusions

In this work, we have investigated the frequency class numbers of codons with respect to the three important classes of error-detecting codes that play a role in the theory of the genetic code: the class COM of all maximal comma-free codes, the class C3 of all maximal self-complementary $C^{3}$ codes and finally, the class CIRC of all maximal self-complementary circular codes. The results show two surprising facts. Firstly, for each of the classes COM, C3 and CIRC, there are only very few frequency class numbers of codons. Secondly, the frequency class numbers yield partitions of the set of codons that become finer when passing from the class COM via the class C3 to the class CIRC (see the Table 7 below for a visualization of this refinement).

The existence of only a few frequency class numbers for each of the classes COM, C3 and CIRC is explained by a mathematical theory using group theory. Moreover, parts of the main Theorems 6–8 are given in the Appendix. The main result, however, is the refinement property shown in the table above. Since the class C3 of maximal self-complementary $C^{3}$ codes is a subclass of the class CIRC of all maximal self-complementary circular codes, the refinement property of the corresponding frequency class numbers is a consequence. However, the first refinement from the class COM of all maximal comma-free codes to the class C3 of all maximal self-complementary $C^{3}$ codes is a surprise. No maximal comma-free code is self-complementary; hence, the two classes COM and C3 (even COM and CIRC) are disjoint. That the frequency class numbers with respect to C3 are still a refinement of the frequency class numbers with respect to COM is a clear hint at a relation between the two classes of codes and supports the theory that the genetic code in its present form evolved from earlier ancient codes in a way that stronger error-detecting and error-correcting properties were weakened to codes that still allow error-detection and error-correction, but in a *less effective* form. Ancient codes used less codons and coded for less amino acids; hence, comma-free codes that detect a frameshift in a reading window of only three bases, hence immediately, could be incorporated. As soon as the genetic codes got more complex involving all codons and coding for a larger number of amino acids, the weaker circular codes that detect frameshifts eventually and in a larger reading window (of 13 nuclear bases) had to take over the error-detection and error-correction function.

## Figures and Tables

**Table 1:** The table shows all non-trivial codons and their frequency class number with respect to maximal comma-free codes. Codons colored in the same color have the same frequency class number.

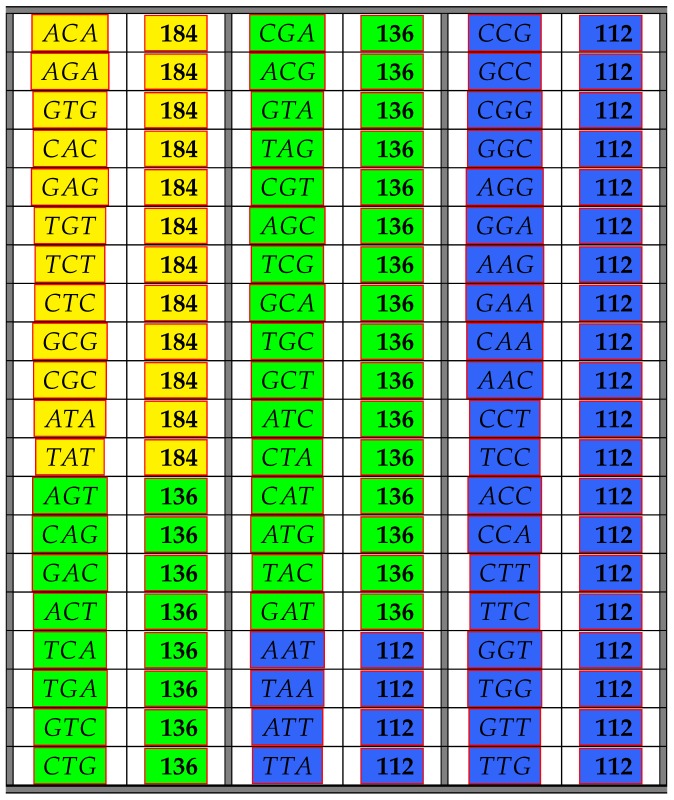

**Table 2:** The table of codons and their frequency numbers with respect to the class COM of all maximal comma-free codes. The class 184 is highlighted in yellow, 112 in blue and 136 in green.

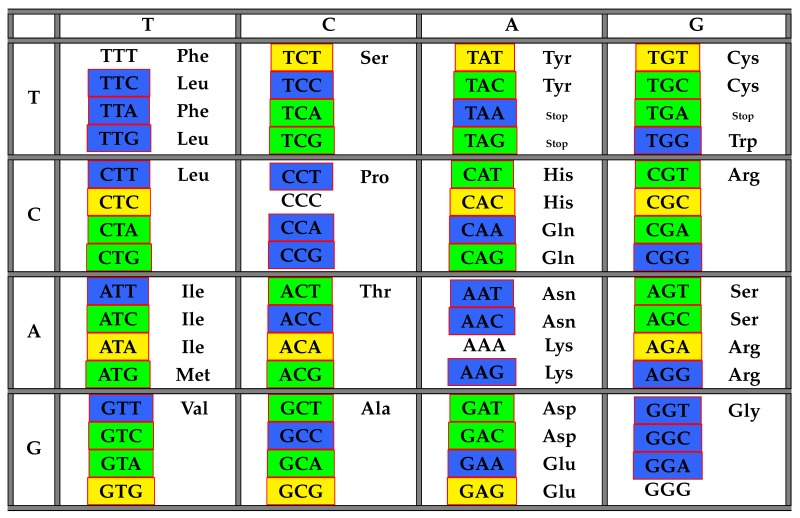

**Table 3:** The table shows all non-trivial codons and their frequency class number with respect to maximal circular self-complementary codes.

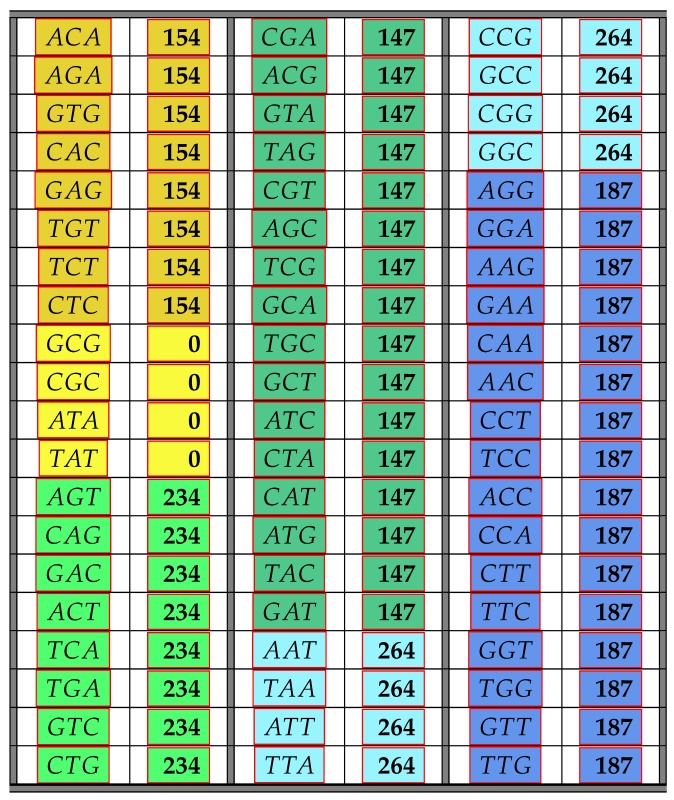

**Table 4:** The table of codons and their frequency numbers with respect to the class CIRC of all maximal circular self-complementary codes. The class 264 is highlighted in light blue, 187 in dark blue, 234 in light green, 154 in light yellow, 147 in dark green and 0 in dark yellow.

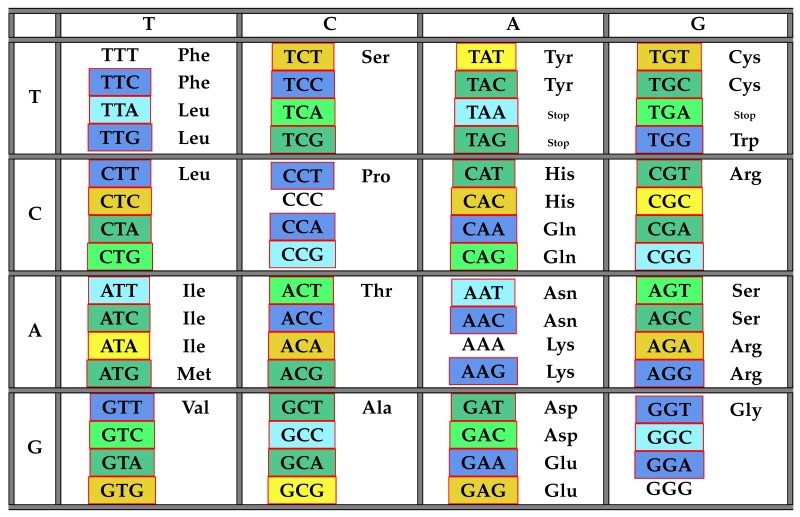

**Table 5:** The table shows all non-trivial codons and their frequency class number with respect to maximal self-complementary $C^{3}$ codes.

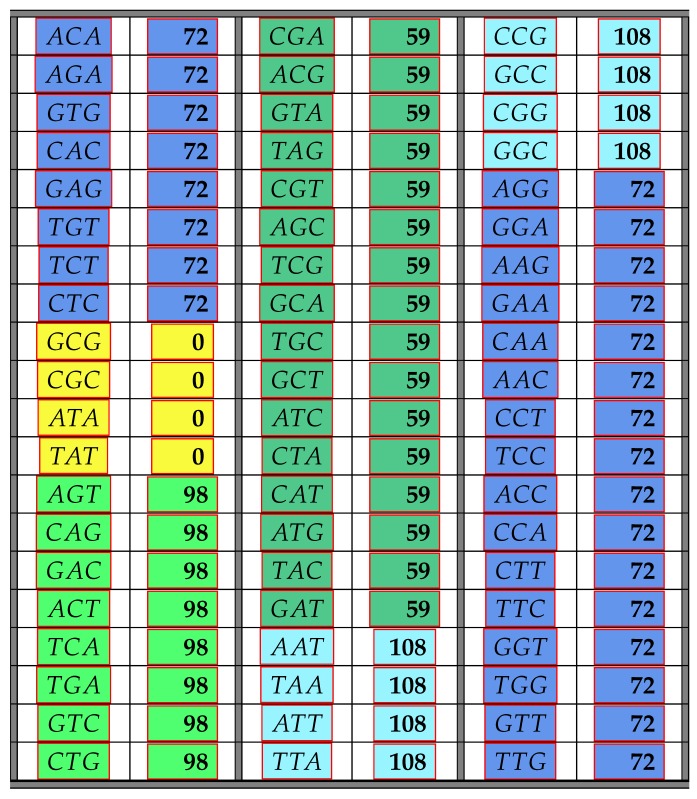

**Table 6:** The table of codons and their frequency numbers with respect to the class C3 of all maximal self-complementary $C^{3}$ codes. The class 108 is highlighted in light blue, 72 in dark blue, 98 in light green, 59 in dark green and 0 in dark yellow.

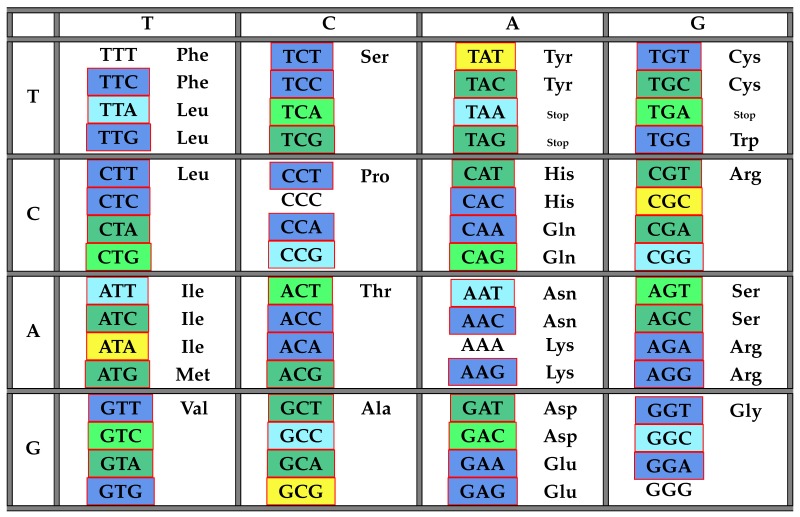

**Table 7:** The table shows all non-trivial codons with their frequency class number with respect to the classes COM, C3 and CIRC.

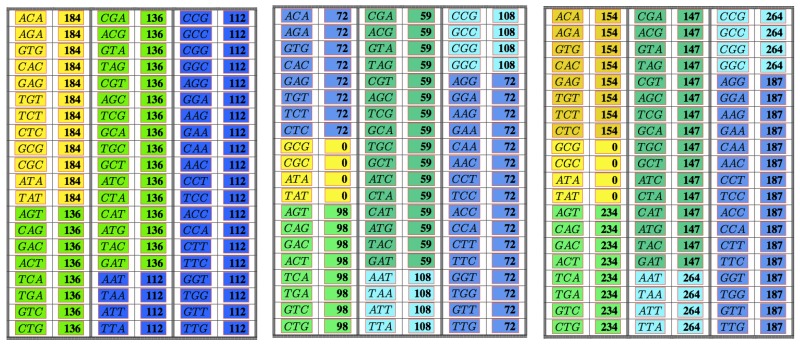

## Appendix A. Proof of Theorem 6

**Proof.** Clearly, any codon $x\in B^{3}\backslash \left\{AAA,CCC,GGG,TTT\right\}$ is either of the form $B_{1}B_{2}B_{1}$, or $B_{1}B_{1}B_{2}$, or $B_{1}B_{2}B_{2}$, or $B_{1}B_{2}B_{3}$ with $B_{i}\in B$ all different, *i.e.*, either the codons consist of three different nucleic bases or two bases are identical. Thus, Cases (1)–(3) of Theorem 6 form a complete case distinction. Moreover, since any bijective transformation $\pi \in S_{B}$, as well as the reversing transformation $\overset{\leftarrow }{}$ preserve comma-freeness, it is obvious that any two codons $x,x^{\prime }\in B^{3}$ must have the same frequency class numbers $u_{\mathrm{COM}}\left(x\right)=u_{\mathrm{COM}}\left(x^{\prime }\right)$, provided they can be mapped onto each other by such transformations. We now first prove that this is indeed the case in all three cases of Theorem 6.

(1) Let $x,x^{\prime }\in B^{3}$ be two codons of the form $x=B_{1}B_{2}B_{1}$ and $x^{\prime }=B_{1}^{\prime }B_{2}^{\prime }B_{1}^{\prime }$ with $B_{1},B_{2},B_{1}^{\prime },B_{2}^{\prime }\in B$, such that $B_{1}\neq B_{2}$ and $B_{1}^{\prime }\neq B_{2}^{\prime }$. Obviously, the bijective transformation $\pi \in S_{B}$ with:
  $$\pi \left(B_{1}\right)=B_{1}^{\prime }\mathrm{and}\pi \left(B_{2}\right)=B_{2}^{\prime }$$
  maps *x* onto $x^{\prime }$.
(2) Let $x,x^{\prime }\in B^{3}$ be two codons of the form $x=B_{1}B_{1}B_{2}$ and $x^{\prime }=B_{1}^{\prime }B_{1}^{\prime }B_{2}^{\prime }$ with $B_{1},B_{2},B_{1}^{\prime },B_{2}^{\prime }\in B$, such that $B_{1}\neq B_{2}$ and $B_{1}^{\prime }\neq B_{2}^{\prime }$. As above, the bijective transformation $\pi \in S_{B}$ with:
  $$\pi \left(B_{1}\right)=B_{1}^{\prime }\mathrm{and}\pi \left(B_{2}\right)=B_{2}^{\prime }$$
  maps *x* onto $x^{\prime }$. The same holds for codons $x,x^{\prime }\in B^{3}$ of the form $x=B_{1}B_{2}B_{2}$ and $x^{\prime }=B_{1}^{\prime }B_{2}^{\prime }B_{2}^{\prime }$. Finally, if $x,x^{\prime }$ are of the form $x=B_{1}B_{1}B_{2}$ and $x^{\prime }=B_{1}^{\prime }B_{2}^{\prime }B_{2}^{\prime }$, then we use the reversing map $\overset{\leftarrow }{}$ concatenated with the following map *σ* to map *x* onto $x^{\prime }$:
  $$\sigma \left(B_{1}\right)=B_{2}^{\prime }\mathrm{and}\sigma \left(B_{2}\right)=B_{1}^{\prime }$$
(3) Let $x,x^{\prime }$ be two codons of the form $x=B_{1}B_{2}B_{3}$ and $x^{\prime }=B_{1}^{\prime }B_{2}^{\prime }B_{3}^{\prime }$ with $B_{i},B_{i}^{\prime }\in B$, such that $B_{1}\neq B_{2},B_{1}\neq B_{3},B_{2}\neq B_{3}$ and $B_{1}^{\prime }\neq B_{2}^{\prime },B_{1}^{\prime }\neq B_{3}^{\prime },B_{2}^{\prime }\neq B_{3}^{\prime }$. Similar to the other two cases, we define the bijective transformation $\delta \in S_{B}$ the following way:
  $$\delta \left(B_{1}\right)=B_{1}^{\prime },\delta \left(B_{2}\right)=B_{2}^{\prime },\mathrm{and}\delta \left(B_{3}\right)=B_{3}^{\prime }$$

We have thus shown that there are only three possible frequency numbers for codons with respect to the class COM of maximal comma-free codes. The exact values of these frequency class numbers have been found by computer calculations. However, for Case (3), we even have a proof. Given a codon $x=B_{1}B_{2}B_{3}\in B^{3}$ with all $B_{i}$ different, it is readily seen that the shifted codons $\alpha_{1}\left(x\right)$ and $\alpha_{2}\left(x\right)$ are of the same form. Thus, we have $u_{\mathrm{COM}}\left(x\right)=u_{\mathrm{COM}}\left(\alpha_{1}\left(x\right)\right)=u_{\mathrm{COM}}\left(\alpha_{2}\left(x\right)\right)$. Equation (5) implies that $u_{\mathrm{COM}}\left(x\right)=408:3=136$. Moreover, there is a relation between the frequency numbers $u_{1},u_{2}$ of codons from Case (1) and Case (2). Given a codon $x=B_{1}B_{2}B_{1}\in B^{3}$ from Case (1) the shifted codons $\alpha_{1}\left(x\right)=B_{2}B_{1}B_{1}$ and $\alpha_{2}\left(x\right)=B_{1}B_{1}B_{2}$ are codons of the form described in Case (2). Thus, it follows that:

$$u_{1}=408-2\cdot u_{2}$$

In particular, if $u_{2}=112$, then $u_{1}=408-2\cdot 112=184$. ☐

## Appendix B. Proof of Theorem 7

**Proof.** It is easy to see that the cases described in Theorem 7 give a complete case distinction for the set of codons $B^{3}\backslash \left\{AAA,CCC,GGG,UUU\right\}$. In fact, any such codon has to be of one of the forms described in (1)–(6). As in the proof of Theorem 6, it suffices to show that codons of the same form can be mapped onto each other by some bijective transformation or the reversing transformation $\overset{\leftarrow }{}$ in order to show that the corresponding frequency class numbers are the same. However, since we are dealing with the class CIRC of all maximal self-complementary circular codes, we need to find bijective transformations that preserve self-complementarity. These permutations have been classified in [12] as a subgroup *L* of the permutation group $S_{B}$. In fact, the group *L* consists of the following eight transformations:

$$\begin{array}{cc} L:= & \{id,c,p,r,\pi_{\mathrm{CG}}:\left(A,C,G,U\right)\mapsto \left(A,G,C,U\right),\pi_{\mathrm{AU}}:\left(A,C,G,U\right)\mapsto \left(U,C,G,A\right),\\ & \pi_{\mathrm{ACUG}}:\left(A,C,G,U\right)\mapsto \left(C,U,A,G\right),\pi_{\mathrm{AGUC}}:\left(A,C,G,U\right)\mapsto \left(G,A,U,C\right)\}. \end{array}$$

where:

$$\begin{array}{c} \text{id}:(A,U,C,G)\to (A,U,C,G)\mathrm{is the identity};\\ \text{SW}\;(\text{or}\;\text{c})\;:(A,U,C,G)\to (U,A,G,C)\mathrm{is the strong/weak}(\mathrm{SW})\mathrm{or complementary transformation};\\ \text{YR}\;(\text{or}\;\text{p})\;:(A,U,C,G)\to (G,C,U,A)\mathrm{is the pyrimidine/purine}(\mathrm{YR})\mathrm{transformation and};\\ \text{KM}\;(\text{or}\;\text{r})\;:(A,U,C,G)\to (C,G,A,U)\mathrm{is the and keto/amino}(\mathrm{KM})\mathrm{transformation}. \end{array}$$

We now show that indeed in any of the cases of Theorem 7, (1)–(6), we can find such a permutation *π* from *L*.

(1) If $x=B_{1}c\left(B_{1}\right)B_{1}\in B^{3}$ and $x^{\prime }=B_{1}^{\prime }c\left(B_{1}^{\prime }\right)B_{1}^{\prime }\in B^{3}$, then either π=id (in case $B_{1}=B_{1}^{\prime }$) or one of the three permutations c,p,r (in case $B_{1}\neq B_{1}^{\prime }$) does the job.
(2) As in Case (1), either the identity id or one of the three permutations c,p,r does the job in combination with the reversing transformation $\overset{\leftarrow }{}$.
(3)–(6) Similarly to Cases (1) and (2), one can define permutations in *L* that do the job.

As in Theorem 6, the exact values of the frequency class numbers have been determined by computer calculations. However, in Case (1), it is also easy to see that the frequency class number is zero. To see this, let $x=B_{1}c\left(B_{1}\right)B_{1},B_{1}\in B$ be a codon in a maximal self-complementary circular code. It follows that also its anticodon $x^{\prime }=c\left(B_{1}\right)B_{1}c\left(B_{1}\right)$ is in the code due to self-complementarity. However, then the word $xx^{\prime }=B_{1}c\left(B_{1}\right)B_{1}c\left(B_{1}\right)B_{1}c\left(B_{1}\right)$ has two decompositions when read on the circle. This contradicts the circularity of the code (compare also [12]). Thus, no codon of the form $B_{1}c\left(B_{1}\right)B_{1}$, $B_{1}\in B$ can be contained in a code from CIRC, and therefore, its frequency class number is zero.

Moreover, as in the proof of Theorem 6, one can show that the frequency class numbers $u_{1}$ - $u_{6}$ from the Cases (1)–(6) of Theorem 7 satisfy the following equations:

u1+2·u2=528,and 2·u3+u4=528 as well as 2·u6+u5=528

In particular, it follows that $u_{2}=\frac{528-0}{2}=264$. ☐

## Appendix C. Proof of Theorem 8

**Proof.** As in the proof of Theorem 7, we can show that the cases in Theorem 8 form a complete case distinction and that the frequency class numbers are the same for codons of the same forms described in (1)–(5) of Theorem 7. The exact values are once more determined by computer calculations. In fact, we have for the frequency class numbers $u_{1}$ to $u_{5}$ from the cases of Theorem 8 that:

2·u1+u2=216 and 3u3=216 as well as u4+2·u5=216

In particular, we have $u_{1}=\frac{216-0}{2}=108$ and $u_{3}=\frac{216}{3}=72$. ☐
